# Supplementary material for: USP25 regulates KEAP1-NRF2 anti-oxidation axis and its inactivation protects acetaminophen-induced liver injury in male mice
Source: Nat Commun. 2023 Jun 20;14:3648. doi: 10.1038/s41467-023-39412-6 (PMC10282087; doi:10.1038/s41467-023-39412-6)
Supplement: Supplementary file 1 — Supplementary Information [file 41467_2023_39412_MOESM1_ESM.pdf]

USP25 Regulates KEAP1-NRF2 Anti-Oxidation Axis and Its Inactivation Protects Acetaminophen-Induced Liver Injury in Male Mice

Changzhou Cai [1, *], Huailu Ma [2, *], Jin Peng [3], Xiang Shen [4], Xinghua Zhen [3], Chaohui Yu [1], Pumin Zhang [2, 3, 5, #], Feng Ji [1, #], and Jiewei Wang [1, #]

[1] Department of Gastroenterology, The First Affiliated Hospital of Zhejiang University School of Medicine, Hangzhou, Zhejiang 310003, China.

[2] Institute of Translational Medicine, Zhejiang University School of Medicine, Hangzhou, Zhejiang 310058, China.

[3] Zhejiang Provincial Key Laboratory of Pancreatic Disease, The First Affiliated Hospital of Zhejiang University School of Medicine, Hangzhou, Zhejiang 310003, China.

[4] Chaser Therapeutics, Inc., Hangzhou, Zhejiang 310018, China

[5] Cancer Center, Zhejiang University, Hangzhou, Zhejiang 310058 China.

Running title: USP25 in APAP-induced liver injury

* These authors contributed equally to this work.

[#] Corresponding authors:

Jiewei Wang, Ph.D. jerrywang075@zju.edu.cn

Feng Ji, Ph.D. jifeng@zju.edu.cn

Pumin Zhang, Ph.D. pzhangbcm@zju.edu.cn

Address Correspondence to: Pumin Zhang, Ph.D.
Provincial Key Laboratory of Pancreatic Diseases
The First Affiliated Hospital
Zhejiang University School of Medicine
Hangzhou, Zhejiang Province 310003
China

E-mail: pzhangbcm@zju.edu.cn.

Supplementary Table 1. Antibody information.

Antibody	Cat No.	Supplier	Purpose
USP25	A7975	ABclonal	1:1000 for WB; 2 μg for IP
KEAP1	10503-2-AP	Proteintech	1:2000 for WB; 2 μg for IP
GAPDH	60004-1-Ig	Proteintech	1:5000 for WB
β-Actin	66009-1-Ig	Proteintech	1:5000 for WB
Lamin B1	12987-1-AP	Proteintech	1:1000 for WB
CYP2E1	19937-1-AP	Proteintech	1:3000 for WB
USP28	17707-1-AP	Proteintech	1:1000 for WB
NRF2	Sc-722	Santa Cruz Biotechnology	1:1000 for WB
UB	Sc-8017	Santa Cruz Biotechnology	1:200 for WB
NRF2	ab62352	Abcam	1:1000 for WB
MKK4	9152S	CST	1:1000 for WB
P-MKK4 (Ser257)	4514P	CST	1:1000 for WB
JNK	ab208035	Abcam	1:2000 for WB
P-JNK (Thr183/Tyr185)	4668P	CST	1:1000 for WB
Rabbit IgG	3900S	CST	2 μg for IP
HA	H6908	Sigma-Aldrich	1:2000 for WB
FLAG	F1804	Sigma-Aldrich	1:2000 for WB
Goat anti mouse (H+L)	115-035-003	Jackson ImmunoResearch Laboratories	1:5000 for WB
Goat anti rabbit (H+L)	111-035-003	Jackson ImmunoResearch Laboratories	1:5000 for WB

Supplementary Table 2. Primer sequences used for RT-PCR.

Gene	Sequence
β-Actin-F	5-GGCTGTATTCCCCTCCATCG-3
β-Actin-R	5-CCAGTTGGTAACAATGCCATGT-3
Keap1-F	5-TGCCCCTGTGGTCAAAGTG-3
Keap1-R	5-GGTTCGGTTACCGTCCTGC-3
Nrf2-F	5-TCTTGGAGTAAGTCGAGAAGTGT-3
Nrf2-R	5-GTTGAAACTGAGCGAAAAAGGC-3
Nqo1-F	5-AGGATGGGAGGTACTCGAATC-3
Nqo1-R	5-AGGCGTCCTTCCTTATATGCTA-3
Gclc-F	5-GGGGTGACGAGGTGGAGTA-3
Gclc-R	5-GTTGGGGTTTGTCCTCTCCC-3
Gclm-F	5-AGGAGCTTCGGGACTGTATCC-3
Gclm-R	5-GGGACATGGTGCATTCCAAAA-3
Usp25-F	5-CAGAAGCACCAGCAGACATTT-3
Usp25-R	5-TGGCATTCTTTGCAGTGAGGA-3

Supplementary Table 3. The sequences used for shRNA.

Gene	Sequence
negative control	5-TTCTCCGAACGTGTCACGT-3
shUSP25-1 (human)	5-TCGATGGTGTTCCTACCT-3
shUSP25-2 (human)	5-GGGAGTACTTGAAGGTAAA-3
shUsp25-1 (mouse)	5-GCACAGAAATAGAGAAATA-3
shUsp25-2 (mouse)	5-GAAGAAACGCTCCGAGTGA-3

Supplementary Table 4. Cloning primers used in the present study.

Gene	Sequence (5’ → 3’)
USP25-SIM/UIM-F	5-GCTCTAGAATGGACTACAAAGACGATGACGACAAGACCGTGGAGCAGAACGTGCT-3
USP25-SIM/UIM-R	5-ATAAGAATGCGGCCGCTAAACTAT TTAATCCCTCCAAACTTCTGT-3
USP25-USP-F	5-GCTCTAGAATGGACTACAAAGACGATGACGACAAGTCTCGAAACCCTTATGATAG-3
USP25-USP-R	5-ATAAGAATGCGGCCGCTAAACTATTTACTGAGACGCTAAAAGCTT-3
USP25-CTD-F	5-CGGAATTCATGGACTACAAAGACGATGACGACAAGAAATTGAGAGAGTCAGAGAC-3
USP25-CTD-R	5-ATAAGAATGCGGCCGCTAAACTATTTATCTTCCATCAGCAGGAGT-3
KEAP1-BTB-F	5-GCTCTAGAATGGACTACAAAGACGATGACGACAAGCAGCCAGATCCCAGGCCTAG-3
KEPA1-BTB-R	5-ATAAGAATGCGGCCGCTAAACTATTTAGACAGCACCGTTCATGAC-3
KEAP1-IVR-F	5-CCGGAATTCCGGATGGACTACAAAGACGATGACGACAAGGGTGCTGTCATGTACCAGAT-3
KEAP1-IVR-R	5-ATAAGAATGCGGCCGCTAAACTATTTAGTGCAGGGTGAGCTCCTC-3
KEAP1-DGR-F	5-CCGGAATTCCGGATGGACTACAAAGACGATGACGACAAGAAGCCCACGCAGGTGATGCC-3
KEAP1-DGR-R	5-ATAAGAATGCGGCCGCTAAACTATTCAACAGGTACAGTTCTGCTG-3

Supplementary Figure Legend

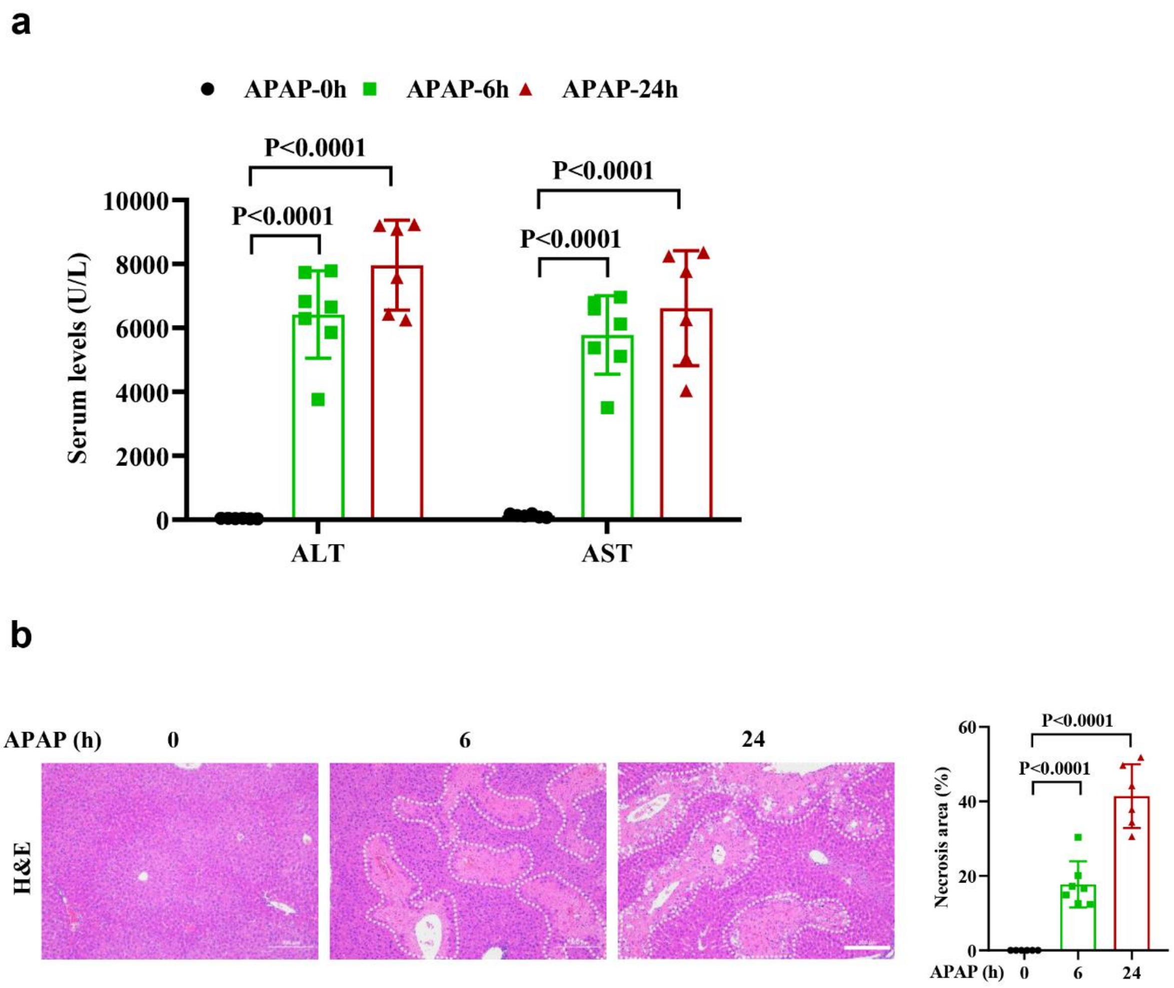

Supplementary Figure 1

Supplementary Fig. 1. Induction of liver injury by APAP administration in mice.

(a) Serum ALT and AST levels in control and APAP-treated mice. Male C57BL/6 mice were fasted for 14 h and then treated with APAP (300 mg/kg, i.p.) for 0, 6, and 24 h. At each time point, 6-7 animals were sacrificed, and the blood and liver samples were collected for analysis. 0 h: n = 6 mice, 6 h: n = 7 mice, 24 h: n = 6 mice.

(b) Hematoxylin and eosin staining of the liver sections of the mice in (a). Necrotic areas were encircled, and the percent of necrotic areas per view field was calculated and plotted. 0 h: n = 6 mice, 6 h: n = 7 mice, 24 h: n = 6 mice. Scale bar, 200 μm.

Error bars denote SEM. Two-tailed student's t tests analysis (a and b). Source data are provided as a Source Data file.

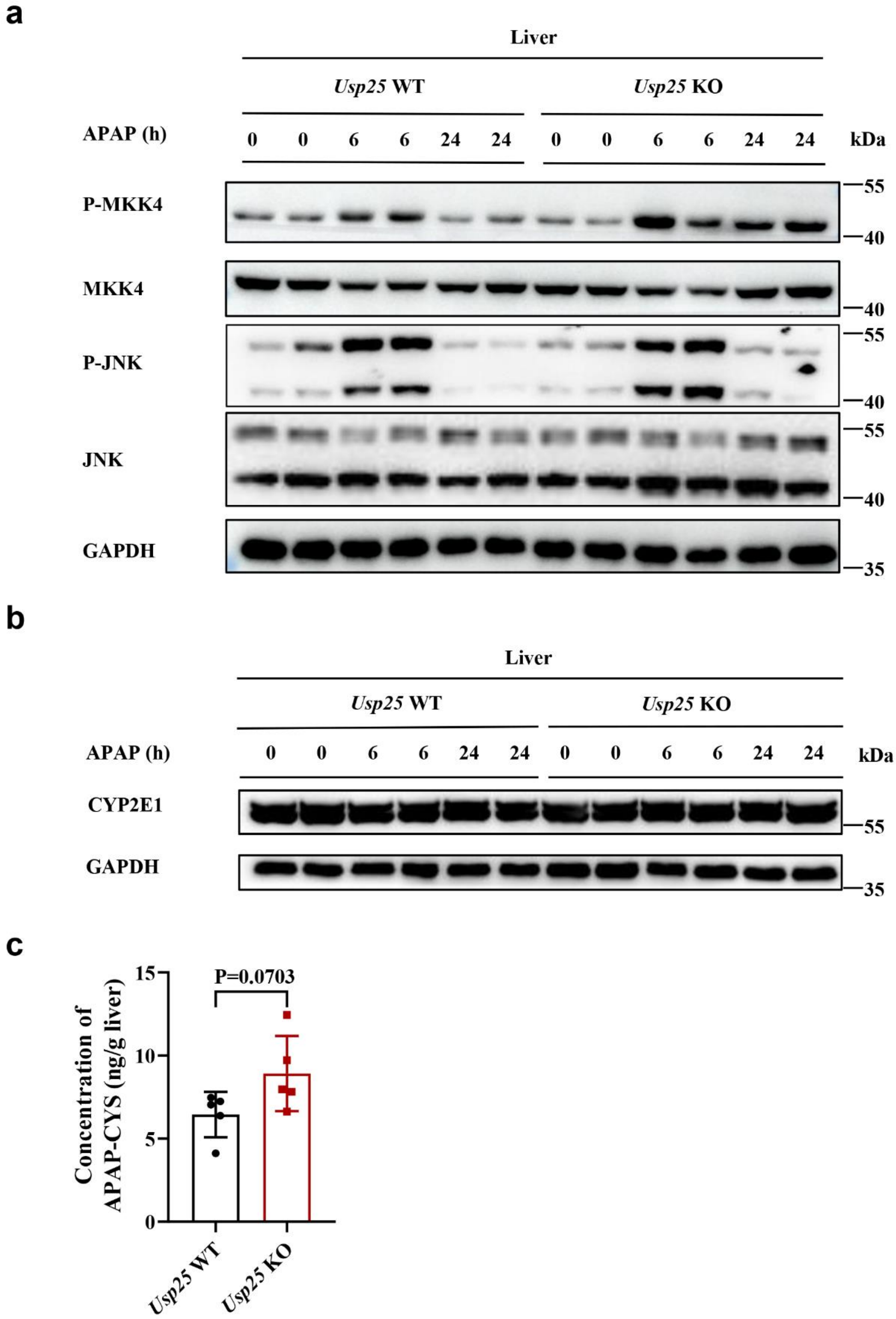

Supplementary Figure 2

Supplementary Fig. 2. The status of *Usp25* does not affect other aspects of APAP-induced responses in the liver.

(a) Western blotting analysis of the indicated proteins in the liver of *Usp25*$^{+/+}$ or *Usp25*$^{-/-}$ mice treated with APAP (300mg/kg, i.p., n = 3-6 per group) for 0, 6 or 24 h.
(b) Western blotting analysis of the protein levels of CYP2E1 in the liver samples from (a).
(c) HPLC coupled mass spectrometry analysis of APAP-CYS concentration in the liver samples from (a). *n* = 5 mice per group. Error bars denote SEM. Statistical significance was analyzed with two-tailed student's t tests analysis. Source data are provided as a Source Data file.

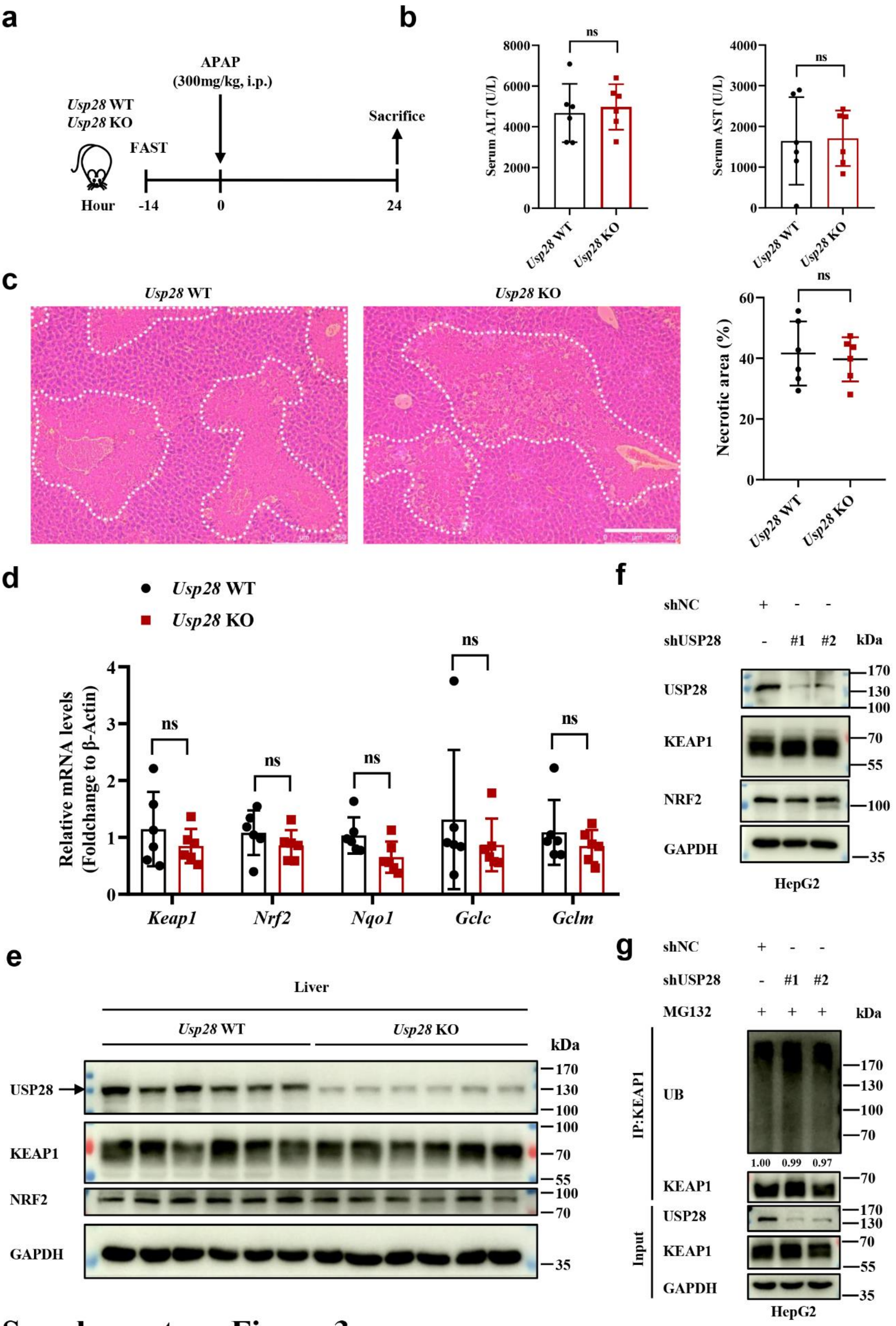

Supplementary Figure 3

Supplementary Fig. 3. The loss of *Usp28* does not provide protection against APAP-induced liver injury.

(a) *Usp28*$^{+/+}$ and *Usp28*$^{-/-}$ mice were treated with APAP (300mg/kg, i.p., n = 6 per group) after 14 h fasting. 24 h after APAP administration, the mice were sacrificed and blood and liver samples were collected for analysis.

(b) Serum ALT and AST levels. *n* = 6 mice per group.

(c) Hematoxylin and eosin staining of the liver sections of the mice in (a). Necrotic areas were encircled, and the percent of necrotic areas per view field was calculated and plotted. *n* = 6 mice per group. Scale bar, 200 µm.

(d) Quantification of *Keap1*, *Nrf2* and NRF2 target gene expression via qPCR in the liver from the mice in (a). *n* = 6 mice per group.

(e) Western blotting analysis of liver proteins from the mice sacrificed at 24 h in (a). The bands with a similar molecular weight to USP28 in the *Usp28*$^{-/-}$ samples are non-specific.

(f) Western blotting analysis of the indicated proteins in HepG2 cells depleted of *USP28* (*n* = 3 biologically independent experiments).

(g) Western blotting analysis of the ubiquitinated species of endogenous KEAP1 in HepG2 cells depleted of *USP28.* The cells were treated with MG132 (20 µM) for 6 h before harvesting for analysis (*n* = 2 biologically independent experiments). The numbers are normalized relative levels of KEAP1 ubiquitination.

Error bars denote SEM. Statistical significance was analyzed with two-tailed student's t tests analysis. Source data are provided as a Source Data file.

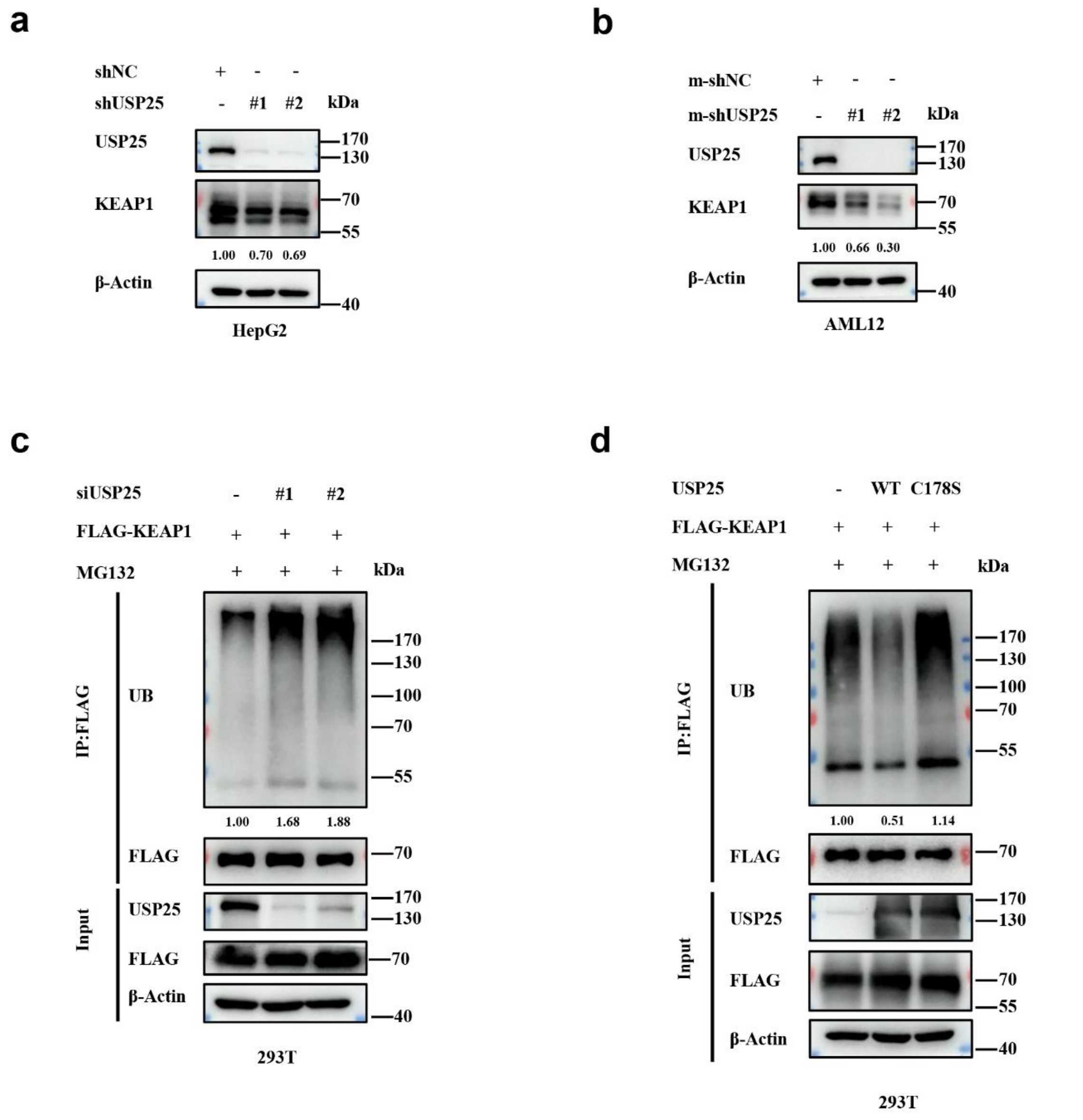

Supplementary Figure 4

Supplementary Fig. 4. USP25 is a deubiquitinase for KEAP1.

(a, b) Western blotting analysis of Keap1 in HepG2 (a) and AML12 (b) cells depleted of *USP25* expression with shRNAs (n = 3 biologically independent experiments).

(c) Determination of the ubiquitination levels of KEAP1 in HEK293T cells depleted of *USP25* expression. FLAG- KEAP1 and siRNAs were co-transfected for 48 h. The cells were treated with MG132 (20 μM) for 6 h before harvesting for analysis (n = 2 biologically independent experiments).

(d) Determination of the ubiquitination levels of KEAP1 in HEK293T cells overexpressing *USP25* or $USP25^{C178S}$. The plasmids for the expression of FLAG- KEAP1, WT USP25 or USP25-C178S were co-transfected for 48 h. The cells were treated with MG132 (20 μM) for 6 h before harvesting for analysis (n = 2 biologically independent experiments).

Source data are provided as a Source Data file.

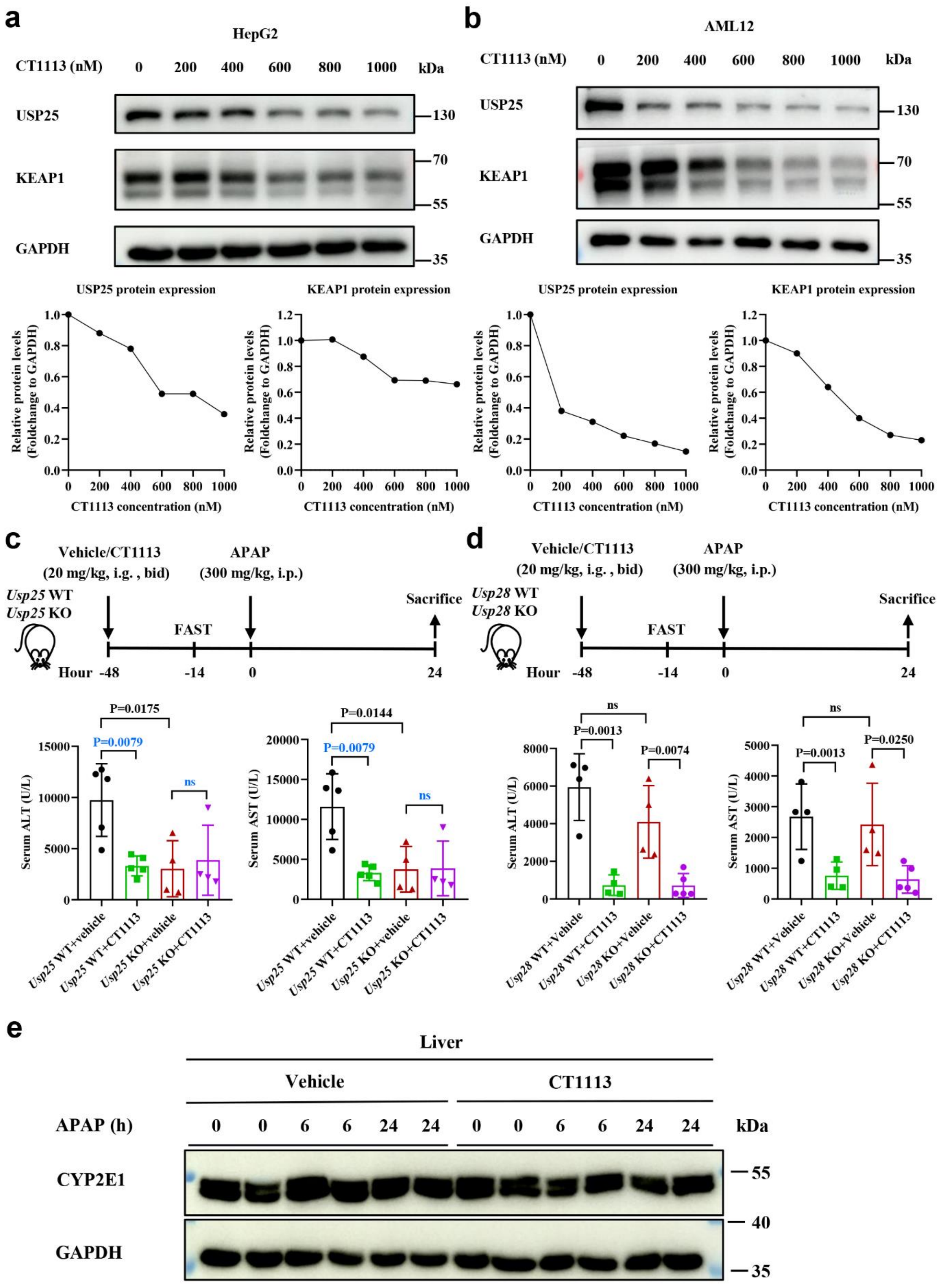

Supplementary Figure 5

Supplementary Fig. 5. CT1113 treatment could not bring additional protection in *Usp25*-deficient mice.

(a, b) Western blotting analysis of USP25 and KEAP1 in HepG2 (a) or AML12 (b) cells treated with CT1113 for 48 h (*n* = 3 biologically independent experiments). The relative protein band intensities were quantified and plotted.

(c, d) *Usp25*$^{+/+}$ and *Usp25*$^{-/-}$ mice (c) or *Usp28*$^{+/+}$ and *Usp28*$^{-/-}$ mice were given vehicle or CT1113 (20 mg/kg, i.g., bid) for 48 h (and continued for the rest of the time until sacrificing), fasted for 14 h, and then given APAP injection (300 mg/kg, i.p.). The animals were sacrificed at 24 h after APAP administration. The blood and liver samples were collected for analyses of serum ALT and AST levels. *Usp25* WT with vehicle: *n* = 5 mice, *Usp25* WT with CT1113: *n* = 5 mice, *Usp25* KO with vehicle: *n* = 4 mice, *Usp25* KO with CT1113: *n* = 4 mice, *Usp28* WT with vehicle: *n* = 4 mice, *Usp28* WT with CT1113: *n* = 4 mice, *Usp28* KO with vehicle: *n* = 4 mice, *Usp28* KO with CT1113: *n* = 5 mice.

(e) Western blotting analysis of the indicated proteins in the liver of the vehicle or CT1113 treated C57BL/6 mice injected with APAP (300mg/kg, i.p., *n* = 3-6 per group) for 0, 6 or 24 h. Error bars denote SEM. Two-tailed student's t tests analysis (c and d), non-parametric tests with blue colored (c). Source data are provided as a Source Data file.

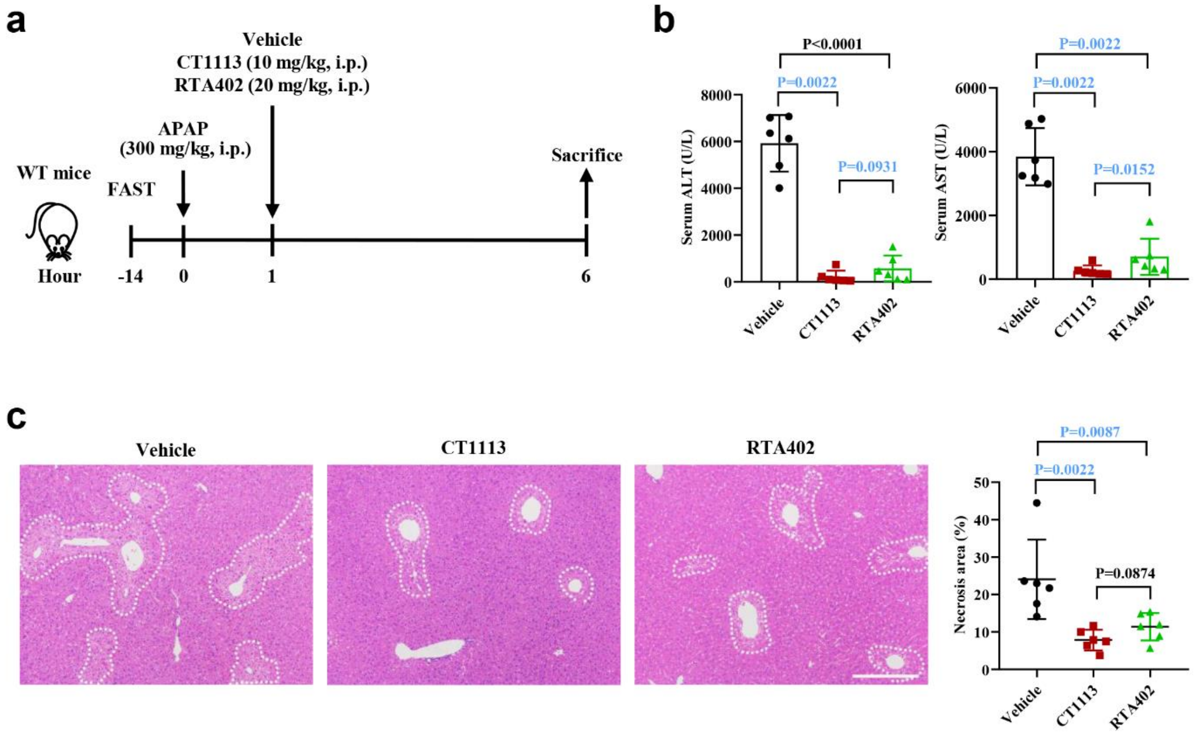

Supplementary Figure 6

Supplementary Fig. 6. Efficacy comparison between CT1113 and RTA402.

(a) Schematic of the experiment. Male C57BL/6 mice were fasted for 14 h, given APAP (300 mg/kg body weight), and CT1113 or RTA402 1 h later for once. Both CT1113 and RTA402 were given through i.p. The animals were sacrificed at 6 h after APAP administration, 6 animals per group.

(b) Serum ALT and AST levels. n = 6 mice per group.

(c) Hematoxylin and eosin staining of the liver sections from mice in (a). Necrotic areas were encircled and quantified. n = 6 mice per group. Scale bar, 200 µm.

Error bars denote SEM. Two-tailed student's t tests analysis (b and c), non-parametric tests with blue colored (b and c). Source data are provided as a Source Data file.
