# Supplementary material for: USP25 regulates KEAP1-NRF2 anti-oxidation axis and its inactivation protects acetaminophen-induced liver injury in male mice
Source: Nat Commun. 2023 Jun 20;14:3648. doi: 10.1038/s41467-023-39412-6 (PMC10282087; doi:10.1038/s41467-023-39412-6)
Supplement: Supplementary file 3 — Description of Additional Supplementary Files [file 41467_2023_39412_MOESM3_ESM.pdf]

Description of Additional Supplementary Files

Supplementary Data 1: This "Supplementary Data" consists of the raw data results for the "Mass spectrometry analysis" section of the manuscript's methods.

The table includes Protein ID, Protein Name, Gene Symbol, Protein Molecular Weight (kDa), Protein Score, Sequence Coverage (%), Number of Specific Peptides, Number of Peptides, Number of MS/MS Spectra, and Abundance.
