# Supplementary material for: USP25 regulates KEAP1-NRF2 anti-oxidation axis and its inactivation protects acetaminophen-induced liver injury in male mice
Source: Nat Commun. 2023 Jun 20;14:3648. doi: 10.1038/s41467-023-39412-6 (PMC10282087; doi:10.1038/s41467-023-39412-6)
Supplement: Supplementary file 5 — Reporting Summary [file 41467_2023_39412_MOESM5_ESM.pdf]

nature portfolio

Corresponding author(s): Pumin Zhang, Feng Ji and Jiewei Wang

Last updated by author(s): May 29, 2023

Reporting Summary

Nature Portfolio wishes to improve the reproducibility of the work that we publish. This form provides structure for consistency and transparency in reporting. For further information on Nature Portfolio policies, see our Editorial Policies and the Editorial Policy Checklist.

Statistics

For all statistical analyses, confirm that the following items are present in the figure legend, table legend, main text, or Methods section.

n/a	Confirmed	
☐	☒	The exact sample size (*n*) for each experimental group/condition, given as a discrete number and unit of measurement
☐	☒	A statement on whether measurements were taken from distinct samples or whether the same sample was measured repeatedly
☐	☒	The statistical test(s) used AND whether they are one- or two-sided<br>*Only common tests should be described solely by name; describe more complex techniques in the Methods section.*
☒	☐	A description of all covariates tested
☐	☒	A description of any assumptions or corrections, such as tests of normality and adjustment for multiple comparisons
☐	☒	A full description of the statistical parameters including central tendency (e.g. means) or other basic estimates (e.g. regression coefficient) AND variation (e.g. standard deviation) or associated estimates of uncertainty (e.g. confidence intervals)
☐	☒	For null hypothesis testing, the test statistic (e.g. *F*, *t*, *r*) with confidence intervals, effect sizes, degrees of freedom and *P* value noted<br>*Give P values as exact values whenever suitable.*
☒	☐	For Bayesian analysis, information on the choice of priors and Markov chain Monte Carlo settings
☒	☐	For hierarchical and complex designs, identification of the appropriate level for tests and full reporting of outcomes
☒	☐	Estimates of effect sizes (e.g. Cohen's *d*, Pearson's *r*), indicating how they were calculated

Our web collection on statistics for biologists contains articles on many of the points above.

Software and code

Policy information about availability of computer code

Data collection	No software and code were used for data collection.
Data analysis	GraphPad Prism version 8; Image J version 1.8.0.

For manuscripts utilizing custom algorithms or software that are central to the research but not yet described in published literature, software must be made available to editors and reviewers. We strongly encourage code deposition in a community repository (e.g. GitHub). See the Nature Portfolio guidelines for submitting code & software for further information.

Data

Policy information about availability of data

All manuscripts must include a data availability statement. This statement should provide the following information, where applicable:

- Accession codes, unique identifiers, or web links for publicly available datasets
- A description of any restrictions on data availability
- For clinical datasets or third party data, please ensure that the statement adheres to our policy

Source data are provided within this paper as a Source Data file. The authors declare that all data supporting the findings described in this paper are available in the article and in the Supplementary Information and from the corresponding author upon reasonable request. Source data are provided with this paper.

Human research participants

Policy information about studies involving human research participants and Sex and Gender in Research.

Reporting on sex and gender	not applicable.
Population characteristics	not applicable.
Recruitment	not applicable.
Ethics oversight	not applicable.

Note that full information on the approval of the study protocol must also be provided in the manuscript.

Field-specific reporting

Please select the one below that is the best fit for your research. If you are not sure, read the appropriate sections before making your selection.

- [x] Life sciences
- [ ] Behavioural & social sciences
- [ ] Ecological, evolutionary & environmental sciences

For a reference copy of the document with all sections, see nature.com/documents/nr-reporting-summary-flat.pdf

Life sciences study design

All studies must disclose on these points even when the disclosure is negative.

Sample size	No statistical method was used to predetermine sample size. For cell-based experiments, n = 3 was chosen based on previous publications in the field (e.g., Nature Communications 2019 Mar 6;10(1):1076). For animal experiments, n ≥ 3 per group was chosen based on previous publications in the field (e.g., Nature Communications 2022 Aug 30;13(1):5111).
Data exclusions	no data were excluded.
Replication	Experiments were repeated with the same conditions and obtained similar results. The numbers of repeats were indicated in figure legends.
Randomization	For in vitro experiments, cells were based on gain or loss of function experiments with appropriate controls. Cells were seeded identically at the oneset of the experiments and randomized into the various treatment groups prior to beginning of treatment protocols. For in vivo analyses, mice were randomly allocated into different treatment groups.
Blinding	Investigators were blinded to analysis whenever it is available.Blinding was used for animal data collection and analysis.

Reporting for specific materials, systems and methods

We require information from authors about some types of materials, experimental systems and methods used in many studies. Here, indicate whether each material, system or method listed is relevant to your study. If you are not sure if a list item applies to your research, read the appropriate section before selecting a response.

Materials & experimental systems

n/a	Involved in the study	
[ ]	[x]	Antibodies
[ ]	[x]	Eukaryotic cell lines
[x]	[ ]	Palaeontology and archaeology
[ ]	[x]	Animals and other organisms
[x]	[ ]	Clinical data
[x]	[ ]	Dual use research of concern

Methods

n/a	Involved in the study	
[x]	[ ]	ChIP-seq
[x]	[ ]	Flow cytometry
[x]	[ ]	MRI-based neuroimaging

Antibodies

Antibodies used	The primary and secondary antibodies used in this study are listed in Supplementary Table 1.<br>USP25 (ABclonal, A7975, 1:1000 for WB; 2 µg for IP);<br>KEAP1 (Proteintech, 10503-2-AP, 1:2000 for WB; 2 µg for IP);<br>GAPDH (Proteintech, 60004-1-Ig, 1:5000 for WB);

β-Actin (Proteintech, 66009-1-Ig, 1:5000 for WB);
Lamin B1 (Proteintech, 12987-1-AP, 1:1000 for WB);
CYP2E1 (Proteintech, 19937-1-AP, 1:3000 for WB);
USP28 (Proteintech, 17707-1-AP, 1:1000 for WB);
NRF2 (Santa Cruz Biotechnology, Sc-722, 1:1000 for WB);
Ub (Santa Cruz Biotechnology, Sc-8017, 1:200 for WB);
NRF2 (Abcam, ab62352, 1:1000 for WB);
MKK4 (CST, 9152S, 1:1000 for WB);
P-MKK4 (Ser257) (CST, 4514P, 1:1000 for WB);
JNK (Abcam, ab208035, 1:2000 for WB);
P-JNK (Thr183/Tyr185) (CST, 4668P, 1:1000 for WB);
Rabbit IgG (CST, 3900S, 2 μg for IP);
HA (Sigma-Aldrich, H6908, 1:2000 for WB);
FLAG (Sigma-Aldrich, F1804, 1:2000 for WB)
Goat anti mouse (H+L) (Jackson ImmunoResearch Laboratories, 115-035-003, 1:5000 for WB)
Goat anti rabbit (H+L) (Jackson ImmunoResearch Laboratories, 111-035-003, 1:5000 for WB)

Validation

The antibodies used in this study are commercially available and validated by the manufacture. Based on the catalog number, the information of these antibody, including validation statements, relevant citations and antibody profiles in online databases, is available in the manufacture's website.
USP25 (ABclonal, A7975, https://abclonal.com.cn/catalog/ A7975);
KEAP1 (Proteintech, 10503-2-AP, https://www.ptgcn.com/products/KEAP1-Antibody-10503-2-AP.htm);
GAPDH (Proteintech, 60004-1-Ig, https://www.ptgcn.com/products/GAPDH-Antibody-60004-1-Ig.htm);
β-Actin (Proteintech, 66009-1-Ig, https://www.ptgcn.com/products/Pan-Actin-Antibody-66009-1-Ig.htm);
Lamin B1 (Proteintech, 12987-1-AP, https://www.ptgcn.com/products/LMNB1-Antibody-12987-1-AP.htm);
CYP2E1 (Proteintech, 19937-1-AP, https://www.ptgcn.com/products/CYP2E1-Specific-Antibody-19937-1-AP.htm);
USP28 (Proteintech, 17707-1-AP, https://www.ptgcn.com/products/USP28-Antibody-17707-1-AP.htm);
NRF2 (Santa Cruz Biotechnology, Sc-722, https://www.scbt.com/p/nrf2-antibody-c-20?requestFrom=search);
Ub (Santa Cruz Biotechnology, Sc-8017, https://www.scbt.com/p/ubiquitin-antibody-p4d1?requestFrom=search);
NRF2 (Abcam, ab62352, https://www.abcam.cn/nrf2-antibody-ep1808y-chip-grade-ab62352.html);
MKK4 (CST , 9152S, https://www.cellsignal.cn/products/primary-antibodies/sek1-mkk4-antibody/9152?site-search-type=Products&N=4294956287&Ntt=9152s&fromPage=plp&_requestid=3900369);
P-MKK4 (Ser257) (CST , 4514P, https://www.cellsignal.cn/products/primary-antibodies/phospho-sek1-mkk4-ser257-c36c11-rabbit-mab/4514?site-search-type=Products&N=4294956287&Ntt=4514p&fromPage=plp&_requestid=3900086);
JNK (Abcam, ab208035, https://www.abcam.cn/jnk1--jnk2--jnk3-antibody-epr18841-95-ab208035.html);
P-JNK (Thr183/Tyr185) (CST, 4668P, https://www.cellsignal.cn/products/primary-antibodies/phospho-sapk-jnk-thr183-tyr185-81e11-rabbit-mab/4668?site-search-type=Products&N=4294956287&Ntt=4668p&fromPage=plp&_requestid=3900164);
Rabbit IgG (CST, 3900S, https://www.cellsignal.cn/products/primary-antibodies/rabbit-da1e-mab-igg-xp-isotype-control/3900?site-search-type=Products&N=102236+4294956287&Ntt=igg&fromPage=plp);
HA (Sigma-Aldrich, H6908, https://www.sigmaaldrich.cn/CN/zh/product/sigma/h6908);
FLAG (Sigma-Aldrich, F1804, https://www.sigmaaldrich.cn/CN/zh/product/sigma/f1804);
Goat anti mouse (H+L) (Jackson ImmunoResearch Laboratories, 115-035-003, https://www.jacksonimmuno.com/catalog/products/115-035-003);
Goat anti rabbit (H+L) (Jackson ImmunoResearch Laboratories, 111-035-003, https://www.jacksonimmuno.com/catalog/products/111-035-003)

Eukaryotic cell lines

Policy information about cell lines and Sex and Gender in Research

Cell line source(s)

HepG2 (HB-8065) and AML12 (CRL-2254) cell lines were obtained from American Type Culture Collection (ATCC, Manassas, VA, USA). HEK293T (GNHu43) cell line were purchased from the Cell Bank of Type Culture Collection of Chinese Academy of Sciences (Beijing, China). Primary hepatocytes were obtained from mice.

Authentication

STR identification.

Mycoplasma contamination

All cell lines tested negative for mycoplasma contemination.

Commonly misidentified lines
(See ICLAC register)

None.

Animals and other research organisms

Policy information about studies involving animals; ARRIVE guidelines recommended for reporting animal research, and Sex and Gender in Research

Laboratory animals

Usp25 knockout mice were obtained from Dr. Chen Dong at Tsinghua University, and Usp28 knockout were generated in the lab. Usp25-/- or Usp28-/- were generated from intercrossing of corresponding heterozygous mice and the Usp25+/+ or Usp28+/+ mice were used as controls in the experiments. C57BL/6J mice (6- to 8-week-old) were purchased from Hangzhou Ziyuan Laboratory Animal Technology Co., Ltd. (Zhejiang, China). All animals were housed in a SPF facility in the First Affiliated Hospital of Zhejiang

	University under temperature (22 ± 2℃) and humidity-controlled (55 ± 5%) conditions with 12-hour light/12-hour dark circadian cycle. They were fed with a standard laboratory diet and had free access to water.
Wild animals	The study did not involve wild animals.
Reporting on sex	We used male mice only.
Field-collected samples	The study did not involve samples collected from the field.
Ethics oversight	All animal experiments were approved by the Animal Care and Use Committee of the First Affiliated Hospital of Zhejiang University.

Note that full information on the approval of the study protocol must also be provided in the manuscript.
